# New use of mononuclear Mn/Co/Ni/Cu complexes of symmetric N_2_O_2_-type Schiff base as hydrogen-evolving catalysts

**DOI:** 10.1098/rsos.260074

**Published:** 2026-10-14

**Authors:** Fatimah Ali Hussein, Ravi Kumar, Matthias Stein, Sandeep Kaur-Ghumaan

**Affiliations:** ^1^ Department of Chemistry, University of Delhi – North Campus, Delhi, India; ^2^ Molecular Simulations and Design Group, Max Planck Institute for Dynamics of Complex Technical Systems, Magdeburg, Germany

**Keywords:** density functional theory studies, homogeneous catalysis, hydrogen, Schiff base ligand, transition metal complexes

## Abstract

Development of sustainable hydrogen production methods is crucial for the future hydrogen economy. Hence, designing cheap, stable and efficient catalysts for hydrogen generation is important. Here, we present the use of the family of eight Schiff base ligand-based transition metal complexes [Mn^III^(Salen)Cl] **1**/[Co^II^(Salen)], **2**/[Ni^II^(Salen)], **3**/[Cu^II^(Salen)], **4**/[Mn^III^(CH_3_Salen)Cl], **5**/[Co^II^(CH_3_Salen)], **6**/[Ni^II^(CH_3_Salen)], **7**/[Cu^II^(CH_3_Salen)] and **8** (Salen/*N*,*N*′-bis(salicylidene)ethylenediamine/H_2_**L^1^**; CH_3_Salen/*N*,*N*′-bis(*o*-hydroxyacetophenone)ethylenediamine/H_2_**L^2^**) as hydrogen evolution reaction (HER) electrocatalysts. Electrochemical studies for the complexes (in CH_3_CN) were carried out in the absence and presence of acids. Catalytic parameters of turnover frequency/overpotential were calculated for the electrocatalytic process. All complexes were found to be efficient HER catalysts in the presence of acids and Co/Ni/Cu complexes were more stable than the Mn complexes. The stability and efficiency order of the catalysts was **2**, **4** > **6** > **7** > **3** > **8** (acetic acid); **1** > **5** (trifluoroacetic acid). Density functional theory studies provided detailed insight into the metal- versus ligand-centred redox transitions and the HER mechanism for all complexes. Successive events of electrochemical reduction (E) and protonation (C) along the HER paths were investigated, and thermodynamically feasible routes were assigned. This work provides insights into the effect of a series of earth-abundant transition metal complex structures, the performance of this series of metal centres on electrocatalysis, and the effect of p*K*_a_ on reactivity. Hence, it will be of value to develop novel and promising catalysts for HER in the future.

## 1. Introduction

Current global energy needs are still predominantly fulfilled by fossil fuels such as coal, natural gas and crude oil, which will be depleted in the near future. Also, as they are non-renewable energy resources, the worldwide change to clean, safe, cost-effective, and renewable energy is urgent. In fact, the use of alternative energy resources needs to increase globally to approximately 50% by 2030 [1,2]. The exploitation of fossil fuels corresponds to higher emissions of CO_2_ into the atmosphere and climate change (global warming). Thus, attention towards alternative energy sources is required for a sustainable environment. Hydrogen is considered to be one of the most promising energy sources for the future as an alternative to traditional fossil fuels [3]. The conventional route for producing hydrogen on an industrial scale involves steam reforming of hydrocarbons, which is a cost-effective process [4]. Other techniques used include electrochemical water splitting, electrolysis, coal and biomass gasification [5]. In nature, the hydrogenase enzymes, utilizing earth-abundant metals such as nickel and iron, are highly efficient for catalysing the production and oxidation of hydrogen [6]. Among the various methods, developing photo-/electro-catalysts for the hydrogen evolution reaction (HER) is a robust approach [7–10]. Inspired by nature, considerable research has been done to develop cheap and effective earth-abundant metal-based molecular electrocatalysts (mostly using organic acids in organic solvents) for use on a large scale to produce clean hydrogen [11–16]. Despite the large number of complexes reported as catalysts for hydrogen generation, obtaining robust and efficient catalysts functional in purely aqueous media still remains a challenge [17,18].

In the past few years, Schiff-base ligand-based complexes have gained a lot of attention, due to their diverse structures (with four coordinating sites and two axial sites open for further binding with ligands), distinctive configurations, structural adaptability, responsiveness to their molecular surroundings, ability to stabilize metals in various oxidation states and useful physical and chemical properties with a wide range of applications—biomedical fields, catalysis, sensors, solar energy devices and processes like photocatalysis and water splitting technologies [7,8,19,20]. The Schiff base ligands can be easily prepared in a single step, and a number of their metal complexes (mono- or multinuclear) have been used as catalysts for various organic reactions [21]. Further, the Schiff base ligand-based complexes with first-row transition metals—manganese, iron, cobalt, nickel, and copper—have also been investigated for catalyst design and improvement [22]. To date, only a few Schiff base metal complexes have been studied as catalysts for hydrogen evolution, in both photo- and electrochemical contexts such as Mn^II^, Fe^II^, Co^II^, Ni^II^ and Cu^II^ complexes with salen, salphen and thiosemicarbazone ligands though there are various reports on CO_2_ reduction and water oxidation (figure 1) [23–31]. Certain systems, like Cu^II^-Schiff base complexes, have been studied for their potential as heterogeneous catalysts in proton reduction reactions (figure 1) [32,33]. Amongst the reported complexes, [Co^II^(Salen)] **2** complex has been shown to act as an HER catalyst under photolytic conditions with a turnover number of 319, and [Cu^II^(Salen)] **4** complex has been reported as an HER catalyst in DMF/acetic acid and under aqueous conditions [30,34]. Though the reported systems with salicylaldehyde-derived donor frameworks, or structurally flexible Schiff base architectures, demonstrate HER activity, there is insufficient understanding available of metal–ligand structure–activity relationships.

**Figure 1.**
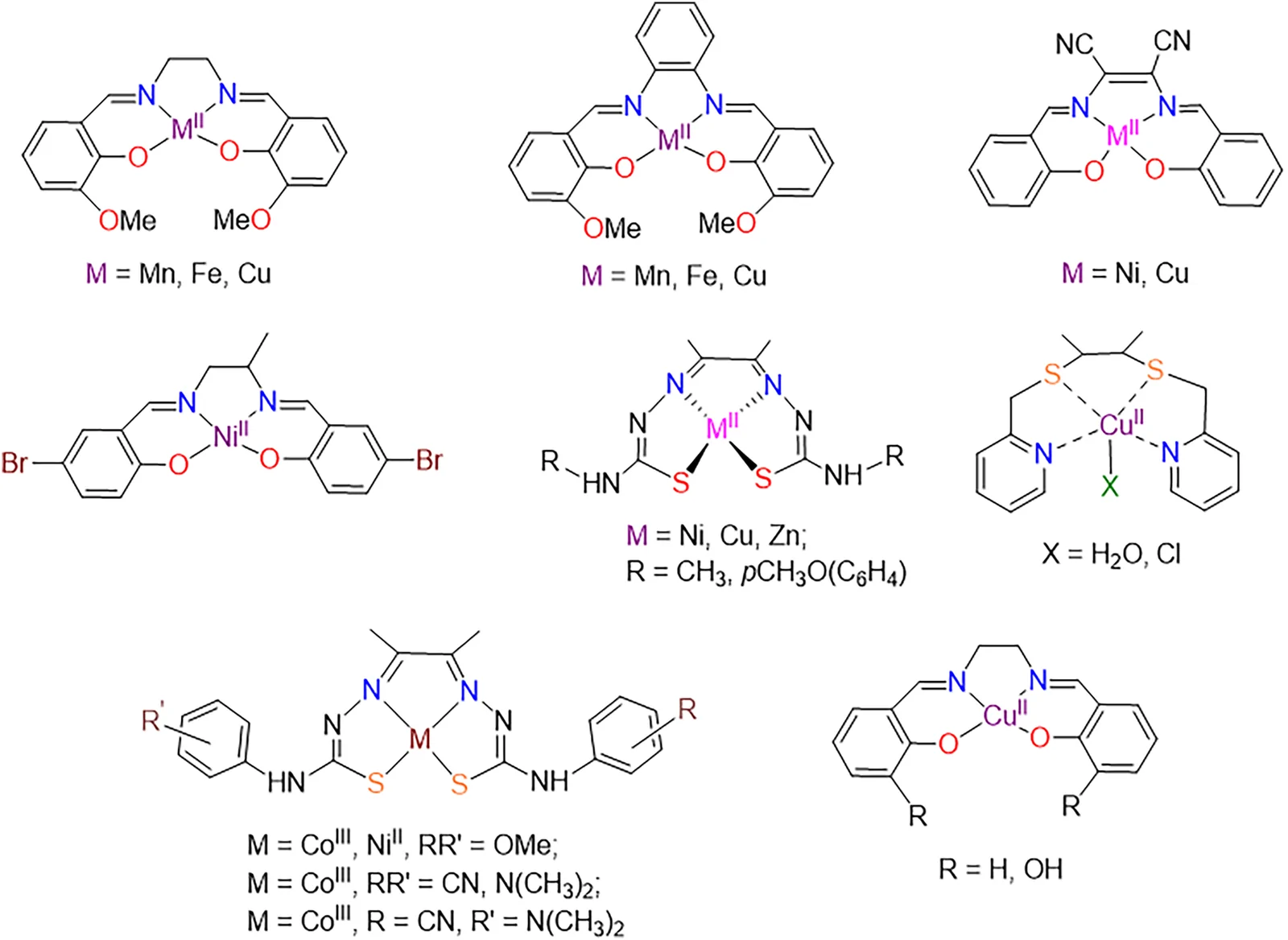
Selected examples of reported N_2_O_2_/N_2_S_2_ [23–33] ligand-based catalysts for electrocatalytic hydrogen generation.

Hence, based on limited reports on the detailed electrochemical behaviour and hydrogen generation mechanism of the Schiff base ligand-based complexes, and the fact that the tetradentate ligands are non-innocent, i.e. both metal centre and ligand can participate in the redox processes, in this work symmetric N_2_O_2_ Schiff base Mn, Co, Ni, and Cu complexes have been investigated for their electrocatalytic hydrogen generation reaction using acetic acid (AcOH) and trifluoroacetic acid (TFA) in acetonitrile in homogeneous solution, due to their reported catalytic activity for various organic reactions [22–26,30,32–35]. In the literature, mostly a single metal centre or a single Schiff base framework is reported, while the present study is a systematic investigation of four first-row transition metals (Mn, Co, Ni, Cu) within the same ligand platform under comparable experimental conditions. The substituent effect on catalytic HER activity is assessed via complexes of both Salen and CH_3_Salen ligands. This work allows for direct evaluation of HER activity, avoiding inconsistencies from different solvents, reference electrodes, or computational methods. The systems studied thus complement and extend the existing literature on Schiff base ligand-based electrocatalysis. Further, though the Ni and Co complexes of the Salen and CH_3_Salen ligands have been reported as HER catalysts, the systems are mainly reported as photo- or electrocatalysts in DMF or DMSO [24,25,33,36].

## 2. Synthesis and characterization of complexes

Mononuclear Mn^III^, Co^II^, Ni^II^ and Cu^II^ complexes **1**–**8** were synthesized using Schiff base ligands (H_2_**L^1^ =** H₂Salen, *N*,*N*′-bis(salicylidene)ethylenediamine; H_2_**L^2^ =** CH_3_Salen, *N*,*N*′-bis(*o*-hydroxyacetophenone)ethylenediamine) and the corresponding metal chloride/metal acetate salts as reported in the literature (scheme 1; see supplementary material) [21,37–44]. Mn^III^ instead of Mn^II^ complexes were obtained when different precursors were used, and various synthesis methods were followed (see supplementary material). The spectroscopic characterization and thermogravimetric analysis (TGA) data matched well with the data reported in the literature [21,40,41,45]. Single-crystal X-ray structures obtained for the complexes showed square planar coordination geometry for Co, Ni, Cu and distorted square pyramidal geometry for the Mn complexes, similar to the structures reported in the literature [30,37,38,46–50].

**Scheme 1.**
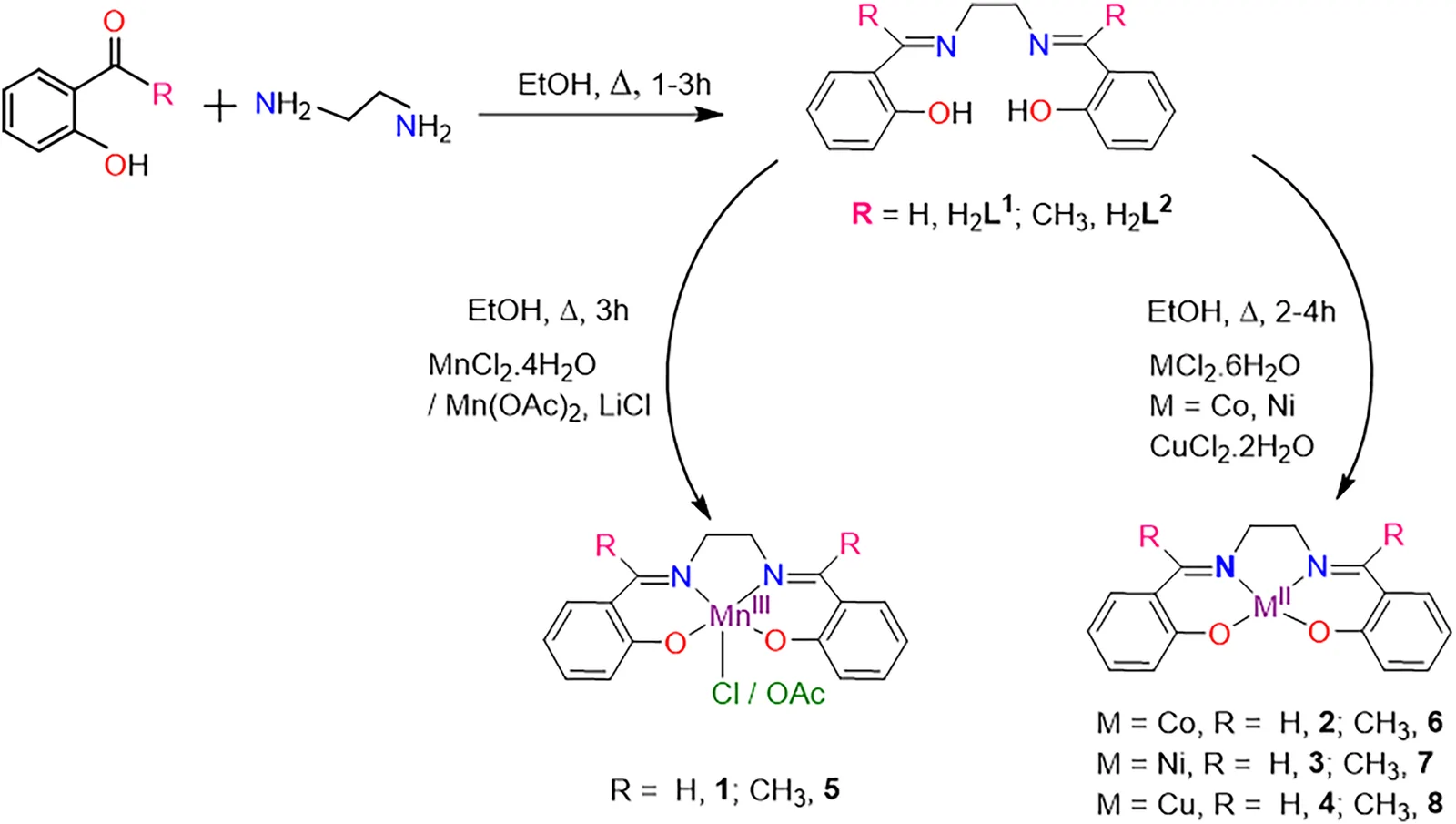
Synthetic scheme for complexes **1**–**8**.

UV–visible spectra of the ligand and the complexes (**1**–**8**) in CH_3_CN are shown in figure S1 of the supplementary material. The free ligand displayed absorption bands between 214-321 nm, which can be assigned as π–π* (transition of the conjugated system) and *n*–π* (transition between the lone pair of electrons on the N-atom and the conjugated phenyl rings) transitions (table 1) [37,41]. On the other hand, the complexes showed peaks between 193-409 nm, which can possibly be assigned to LMCT (from the phenolate to metal, ^1^A_1g_ → ^1^E_g_ transition, characteristic for a square planar geometry), overlapping with *n*–π* and π –π* bands and d–d electronic transitions of the metal orbitals (table 1) [54]. The lack of any observed d–d bands >500 nm, which may be masked by the high-intensity bands, implies a four-coordinate environment for the nickel centre [55]. Overall, our UV–visible spectra confirm the successful synthesis of these eight transition metal Schiff base complexes. Minor shifts in spectral positions may originate from the use of different solvents (pure acetonitrile in this work versus MeOH, DMSO, DMF or mixed solvents reported in the references in table 1).

**Table 1.** UV–visible data for the Schiff-base ligands (H_2_**L^1^**/H_2_**L^2^**) and complexes **1**–**8** in CH_3_CN. The table also includes previously reported data in different solvents for the ligands and complexes.

compound	wavelength (nm)	ref.
**1-**Mn	409, 393, 273(sh), 222, 195	this work
**2-**Co	390, 252, 227(sh), 193	this work
	489, 403, 346^a^	[51]
**3-**Ni	406(sh), 338, 255(sh), 245, 196	this work
	528, 440, 405, 327^b^	[52]
**4-**Cu	359, 272, 244(sh), 228, 196	this work
	575, 374, 282^c^; 560, 356, 268, 244^d^	[30]
**5-**Mn	409(sh), 322(sh), 278, 219(sh), 204	this work
	540, 380, 260, 219^e^	[43]
**6-**Co	383, 252, 225, 193(sh)	this work
	525, 414, 330, 248^c^	[53]
**7-**Ni	404(sh), 327, 251, 194(sh)	this work
**8**-Cu	354, 272, 235, 197	this work
	556, 388, 350^c^	[44]
H_2_**L^1^**	314, 255, 214	this work
	409, 320, 280^f^	[37]
H_2_**L^2^**	317, 253, 216	this work
	386, 327^g^; 390, 336, 232^c^	[38]

^a^CH_2_Cl_2_–CH_3_CN; ^b^EtOH; ^c^DMF; ^d^H_2_O; ^e^CH_2_Cl_2_; ^f^DMSO; ^g^MeOH–H_2_O.

The morphology and particle size of the metal–salen complexes (**1**–**8**) have also been investigated by field emission scanning electron microscopy (FESEM; supplementary material, figures S2 and S3). FESEM images of the obtained complexes show a uniform matrix, indicating homogeneous phase material. The morphology and the average particle size are similar to those of previously reported analogous Schiff base complexes [56].

## 3. Electrochemistry

Electrochemical investigations for the salen ligands (H_2_**L^1^** and H_2_**L^2^**) and their first-row transition metal complexes **1**–**8** were conducted under an argon atmosphere in an acetonitrile solution of 0.1 M tetrabutylammonium hexafluorophosphate (*^n^*Bu_4_NPF_6_) as supporting electrolyte using a glassy carbon working electrode. Irreversible reduction and oxidation peaks were observed at −2.31, 0.99 V (H_2_**L^1^**), −2.35, 0.96 V (H_2_**L^2^**) for the two metal-free salen ligands (supplementary material, figure S6) [57]. The salen ligand-based complexes showed reductions at −0.64, −2.05 (**1**), −1.55, −1.74 (**2**), −2.13 (**3**), −1.69 V (**4**) whereas the CH_3_ salen ligand-based complexes showed reduction peaks at −0.71, −2.16 (**5**), −1.64, −1.87 (**6**), −2.23 (**7**), −1.84 V (**8**) (supplementary material, figures S4 and S5). The complexes showed one-electron quasi-reversible reductions (Δ*E*_p_ > 60 mV). The Co complexes also showed a second reduction at −1.74 (**2**), −1.87 V (**6**). The reduction potentials for the salen complexes were lower than those of the corresponding CH_3_ salen complexes. The reduction potentials for the M^II^ complexes followed the order **2** < **4** < **3** and **6** < **8** < **7**, that is, Co < Cu < Ni. One or two quasi-reversible oxidation peaks were also observed for the complexes between 0.4 and 0.82 V (supplementary material, figure S4). The reductions observed for the complexes can be attributed to the redox processes Mn^III^Mn^II^, Mn^II^Mn^I^; Co^II^/Co^I^, Co^I^Co^0^; Ni^II^ /Ni^I^; Cu^II^/Cu^I^, Cu^I^/Cu^0^ while the oxidations indicate Mn^III^Mn^IV^, Co^II^/ Co^III^, Ni^II^ /Ni^III^ and ligand-based reduction/oxidation [39,48] redox processes. The linear dependence of the current response of the redox events on the square root of the scan rate was indicative of a diffusion-controlled process, which suggests that the electrochemically active species is freely diffusing in the solution. This further prompted possible usage of the complexes as electrocatalysts for hydrogen evolution (supplementary material, figure S7).

To rationalize the electrochemical process of Mn, Co, Ni and Cu Schiff base complexes, we calculated their reduction potential values utilizing density functional theory (DFT). The chosen optimization in solvent with a BP86/def2-TZVP (CPCM) approach has been shown to give reliable calculated reduction potentials for transition metal complexes. For complex **1**, the Mn^III^ to Mn^II^ reduction potential is computed to be −0.58 V, which is in excellent agreement with the first reduction observed experimentally for this complex. The calculated M^II^ to M^I^ (M = Mn, Co, Ni, Cu) reduction potential values (*E*^red^) for Mn, Co, Ni and Cu Schiff base complexes are −2.06 V (**1**), −1.60 V (**2**), −2.11 V (**3**) and −1.70 V (**4**), respectively, corresponding to metal reduction. These values are relative to Fc/Fc^+^ and are in excellent agreement with the above experimental values, as reported in table 2.

**Table 2.** Experimental and calculated reduction potentials for complexes **1**–**8**.

complex	*E*_pc_^red^ (V) (exp)	*E*_pc_^red^ (V) (calc)
**1**, **L^1^**Mn	–0.64, –2.05	–0.58, –2.06
**2**, **L^1^**Co	–1.55, –1.74*	–1.60
**3**, **L^1^**Ni	–2.13	–2.11
**4**, **L^1^**Cu	–1.69	–1.70
**5**, **L^2^**Mn	–0.71, –2.16	–0.75, –2.19
**6**, **L^2^**Co	–1.64, –1.87*	–1.74
**7**, **L^2^**Ni	–2.23	–2.16
**8**, **L^2^**Cu	–1.84	–1.94

(**1**, **5**) Mn^III^L + e^−^ → Mn^II^L and Mn^II^L + e^–^ → Mn^I^L.

(**2**, **6**) Co^II^L + e^–^ → Co^I^L and *Ligand reduction.

(**3**, **7**) Ni^II^L + e^–^ → Ni^I^L.

(**4**, **8**) Cu^II^L + e^–^ → Cu^I^L.

After the modification of hydrogen (–H) to methyl (–CH_3_) at the Schiff base position, the calculated *E*^red^ for complexes **5** (Mn), **6** (Co), **7** (Ni) and **8** (Cu) increases to −2.19 V, −1.74 V, −2.16 V and −1.94 V, respectively, and closely follows the experimental trend (see table 2). The increase in the *E*^red^ values is due to the electron-donating nature of the methyl group, which tends to facilitate the reduction and provide more negative reduction potential values. We have also computed the second reduction potentials for the Co Schiff base complexes, as seen in the CV spectra. However, this second peak is not metal-based but rather corresponds to a ligand-centred reduction [58]. Thus, the second reduction peak at −1.74 V (−1.87 V) for Co^II^ Schiff base complexes corresponds to a ligand radical in complex with Co^II^ (Co^II^-L^•−^), which will be anti-ferromagnetically coupled, explaining the observed peak position [59]. The GGA functional BP86 failed to give the anti-ferromagnetically coupled broken-symmetry state (BS) as the global minimum, whereas the hybrid functional PBE0 yielded a slightly lower-energy BS solution, consistent with the experimentally observed second electrochemical peak at −1.74 V (−1.87 V) for cobalt complexes **2** and **6** (see figure 2).

**Figure 2.**
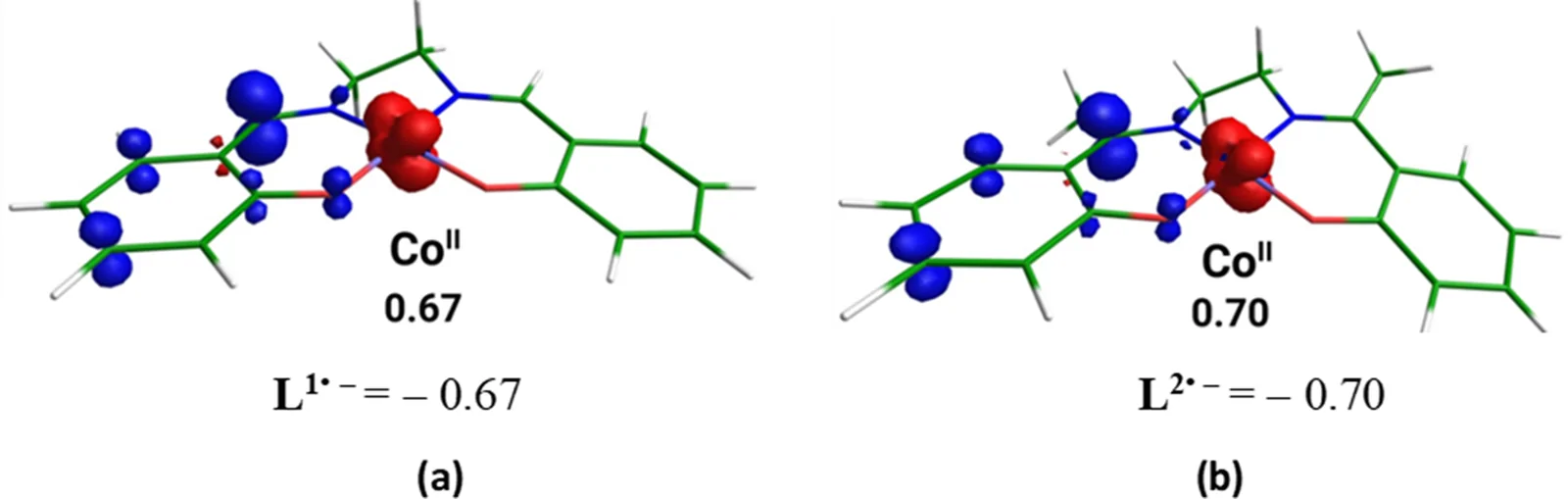
PBE0-D3BJ/def2-TZVP optimized structures of mono-reduced **2** and **6** complexes and spin-density plots of the anti-ferromagnetically coupled states of (a) Co^II^-**L^1^**^• −^ (**2**) and (b) Co^II^-**L^2^**^• −^ (**6**) at 0.01 a.u. contour value.

We found that the unpaired spin density on the doubly reduced species of Co^II^ → Co° complex is merely 0.05 (**2**) (0.07 for **6**) on the metal atoms, and the remaining 0.95 (0.93) spin density, respectively, is distributed over the Schiff base ligand. This indicates that the second reduction step is also ligand-centred (see supplementary material, figure S21).

Complexes **1** and **5** have an intermediate-spin (*S* = 3/2) for Mn^II^ (d^5^) ground state with three unpaired electrons, while other complexes (**2**–**4**, **6**–**8**) possess a low-spin state as the ground state, which is consistent with the literature [60–63]. Co^II^ (d^7^) (**2** and **6**) and Cu^II^ (d^9^) (**4** and **8**) contain only one unpaired electron (*S* = ½), and Ni^II^ (d^8^) (**3** and **7**) refers to closed-shell species. The Mn^II^ spin-density plots correspond to three unpaired electrons, based on the shape of the spin density (see figure 3a,b) [64]. The unpaired electrons in complexes Co^II^ **2** (**6**) and Cu^II^ **4** (**8**) are located in different orbitals, which may affect the hydrogen evolution mechanism. The Co^II^ **2** (**6**) has an unpaired electron in d_*xz*/*yz*_ orbital with a spin density of 0.914 (0.916), which indicates a metal-localized unpaired electron. In contrast, Cu^II^ **4** (**8**) has an unpaired electron in the d_*x*^2^-*y*^2^_ orbital with a spin density of 0.488 (0.485), which indicates a partially delocalized unpaired electron density that incorporates the ligand (see figure 3).

**Figure 3.**
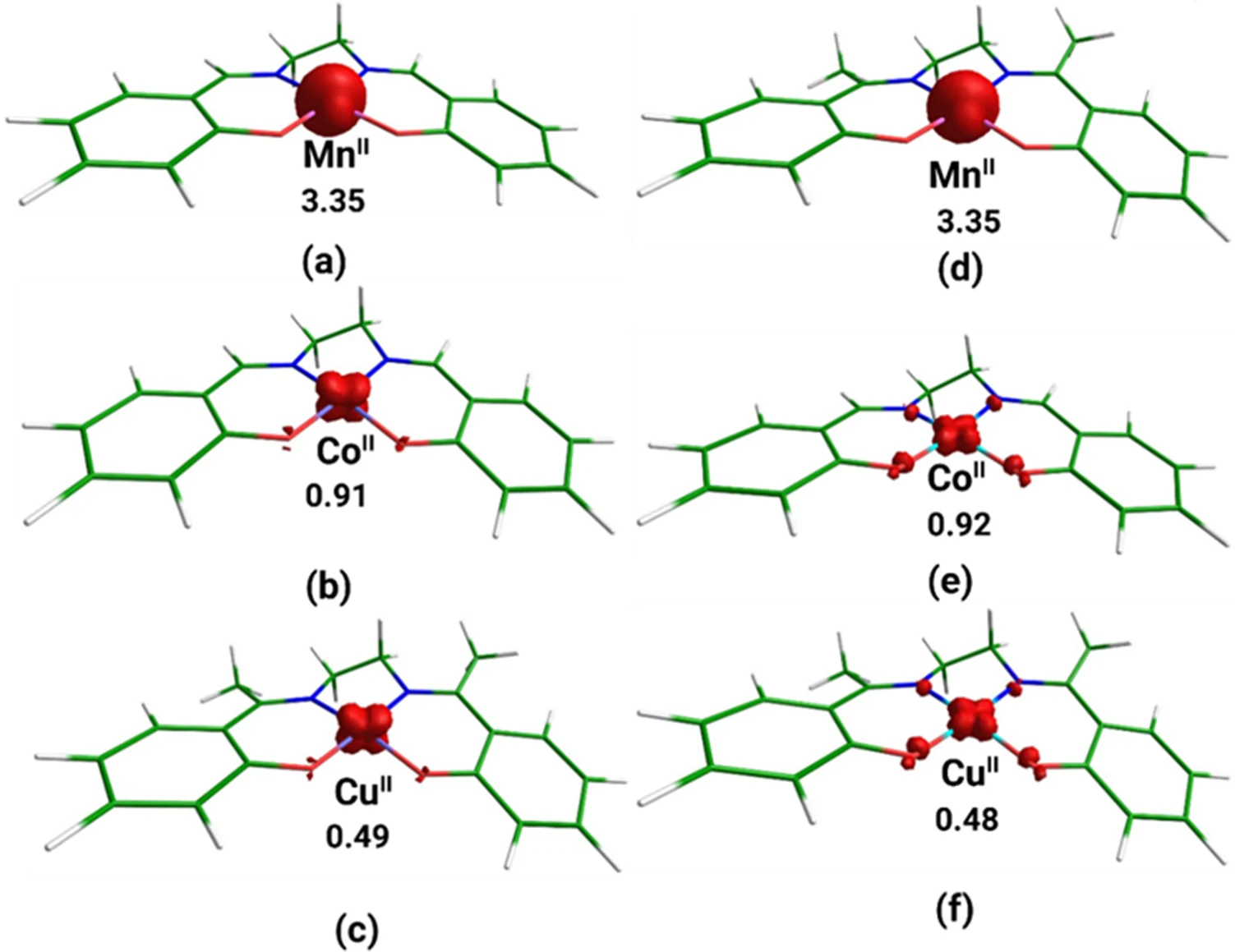
Calculated spin densities of the ground states of (a,d) Mn^II^ (**1**, **5**), (b,e) Co^II^ (**2**, **6**) and (c,f) Cu^II^ (**4**, **8**) Schiff base complexes at 0.03 a.u. isocontour value.

CVs for the electrocatalytic activity of the complexes Mn, Co, Ni, Cu (1 mM) were experimentally obtained upon addition of known concentrations of acetic acid (AcOH; p*K*_a_ = 23.51, *E*^0^ = −1.46 V, CH_3_CN) in acetonitrile (figures 4–7; supplementary material, figure S8) [65–67]. The appearance of irreversible reduction waves, a sharp increase in electric current, cathodic current enhancement and cathodic shifting of the peaks with the addition of acid indicated a catalytic proton reduction.

**Figure 4.**
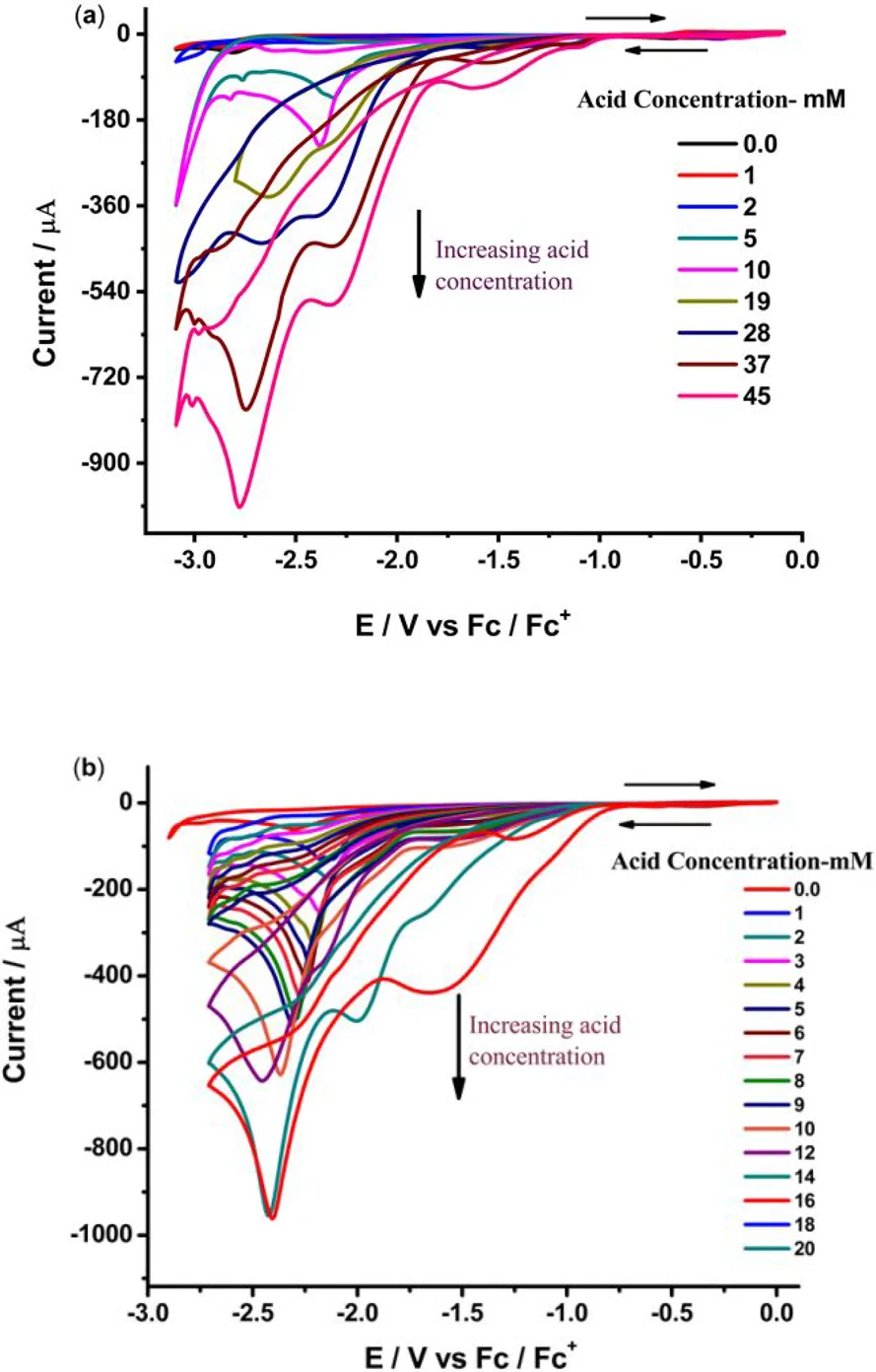
CVs for Mn complexes (1 mM) (a) **1** in the absence and with increasing concentrations of TFA (1–45 mM) and (b) **5** in the absence and with increasing concentrations of TFA (1–20 mM) in CH_3_CN at a scan rate of 0.1 V s^‒1^.

**Figure 5.**
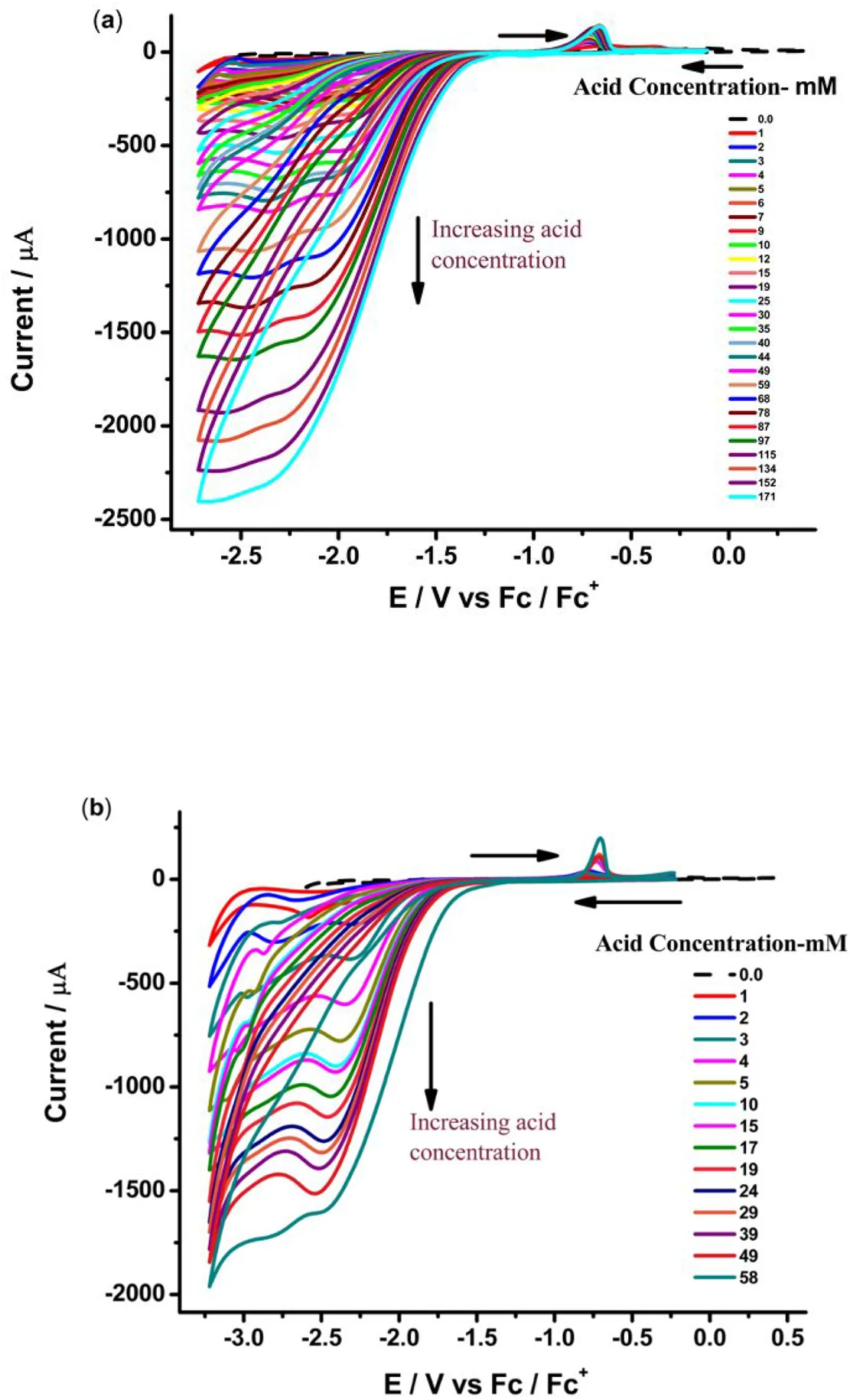
CVs for Co complexes (1 mM) (a) **2** in the absence (**- - -**) and with increasing (—) concentrations of AcOH (0.9–171 mM) and (b) **6** in the absence (**- - -**) and with increasing (—) concentrations of AcOH (0.9–58 mM) in CH_3_CN at a scan rate of 0.1 V s^‒1^.

**Figure 6.**
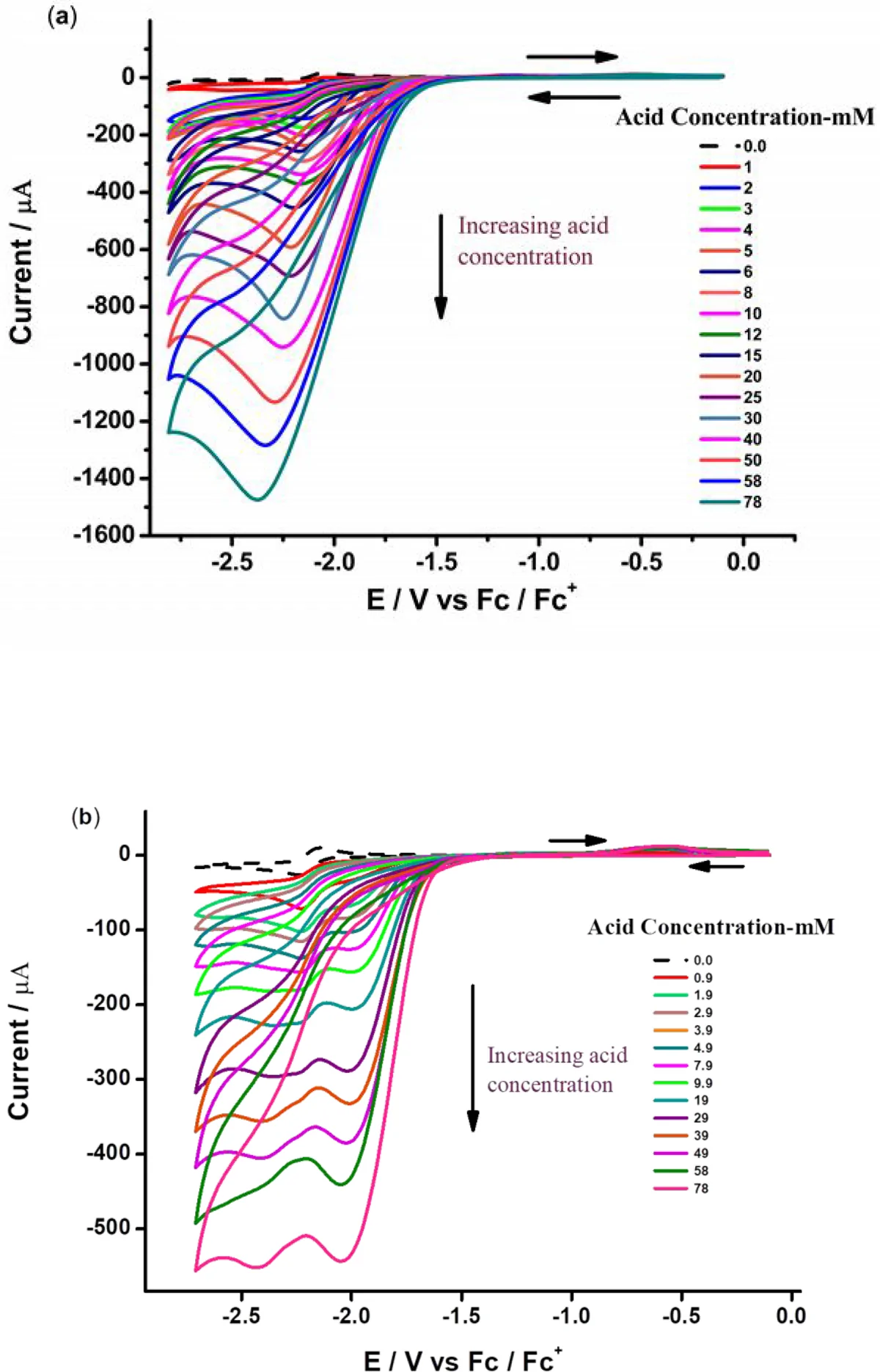
CVs for Ni complexes (1 mM) (a) **3** and (b) **7** in the absence of acid (**- - -**) and with increasing concentrations (—) of AcOH (1–78 mM) in CH_3_CN at a scan rate of 0.1 V s^‒1^.

**Figure 7.**
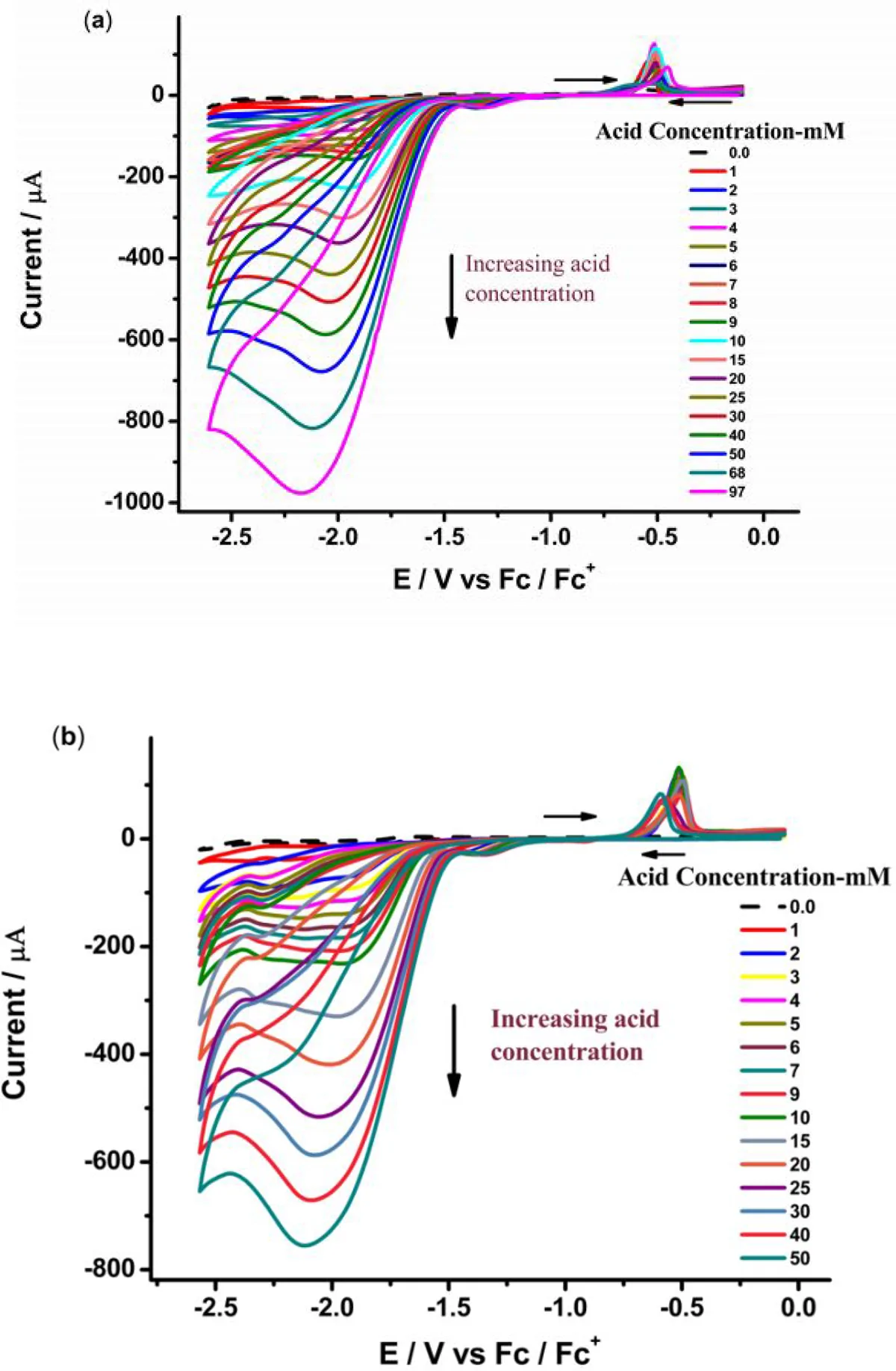
CVs for Cu complexes (1 mM) (a) **4** in the absence (**- - -**) and with increasing (—) concentrations of AcOH (1–97 mM) and (b) **8** in the absence (**- - -**) and with increasing (—) concentrations of AcOH (1–50 mM) in CH_3_CN at a scan rate of 0.1 V s^‒1^.

Further, the appearance of the irreversible catalytic peaks and enhancement of the current of the catalytic peaks at reduction potentials close to the first redox potential of the complexes could be attributed to a proton-coupled electron transfer process leading to the metal-hydride intermediate [22–25]. In addition, a second reduction wave with a cathodic shift of the reductions and increased intensity of the peak current on increasing the amount of acid was also observed for the complexes [22]. The peaks levelled off upon addition of 10 (**1**), 171 (**2**), 78 (**3** and **7**), 97 (**4**), 50 (**5**), 58 (**6**), and 50 mM (**8**) of acetic acid. Though Co, Ni and Cu complexes showed catalytic activity with weak acid (AcOH), the Mn complexes (**1**, **5**) showed poor catalytic activity with weak acids as the proton source. Hence, the performance of complexes **1** and **5** as HER catalysts was further explored in the presence of trifluoroacetic acid (TFA). Addition of TFA to **1** and **5** resulted in two irreversible waves with increasing peak currents on further addition of acid, thus indicating electrocatalytic proton reduction (figure 4). Further, Co, Ni and Cu complexes were not stable in the presence of TFA (p*K*_a_ = 12.7, *E*^0^ = −0.89 V, CH_3_CN), forming colourless solutions on addition of ∼9–14 mM of acid (TFA) [65]. For comparison purposes, the direct reduction of acetic acid was measured under the same experimental conditions but in the absence of the catalysts. In the absence of catalysts, in acetonitrile/AcOH/TFA, the CVs showed negligible current (−1.0 to −3.0 V) at the glassy carbon electrode, thus indicating that the currents observed in the presence of catalysts were indeed due to catalytic proton reduction (supplementary material, figures S9–S11).

A linear plot for *i*_cat_/*i*_p_ versus [AcOH] suggested a first-order dependence on the proton concentration (supplementary material, figure S12). At higher concentrations, the deviation from linearity could be due to decomposition of the catalyst. The catalysts were active towards proton reduction with an *i*_cat_*/i*_p_ ratio (various concentrations of acid) of complexes (Co, Ni and Cu), corresponding to *k*_obs_ (s^−1^) values (608, **2**; 674, **6**; 53, **3**; 80, **7**; 496, **4**; 686, **8**) of the complexes ( table 3; supplementary material, figures S12 and S13) [65–67]. However, the *i*_cat_/*i*_p_ values represent only an estimate of *TOF* (turnover frequency) as steady-state conditions were still not achieved at this point. As observed from the *k*_obs_ values, the Schiff base complexes were moderate to highly efficient as catalysts (Cu > Co > Ni > Mn; supplementary material, figures S12 and S13). However, complexes **2** (Co**L^1^**), **4** (Cu**L^1^**) and **8** (Cu**L^2^**) (**4** < **2** < **8** < **7** < **3** < **6** < **1** < **5**) showed the lowest overpotentials and complexes **2**, **8** and **6** showed high *TOF* (**8**> **6** > **2** > **4** >**7** > **3**> **5** > **1**) in comparison to Ni and Mn complexes. The *TOF* values of the Schiff base complexes were calculated using equations (3.1) and (3.2):

(3.1)icat/ip=n/0.446RTkobs/Fv,(3.2)kobs=k1[H+],

where *i*_cat_ is the peak current in the presence of acid, *i*_p_ is the peak current in the absence of acid, *n* is the number of electrons involved in the catalytic process, *k*_obs_ (turnover frequency, *TOF*, s^−1^) is the observed rate constant, *R* is the ideal gas constant, *T* is temperature in kelvin (298 K), *F* is Faraday's constant, and *v* is the scan rate. A scan rate of 0.1 V s^−1^ was used to obtain the values for *TOF*. As observed, at scan rates 0.1 V s^−1^ there was not much increase in *i*_cat_ and, hence, equations (3.1) and (3.2) can be used to calculate the *TOF* [65–67]. Although equations (3.1) and (3.2) signify pseudo-first-order systems, these equations can also be used to determine *TOF*s for more complicated systems for comparison with other catalysts. Tafel plots are shown in figure S20 of the supplementary material.

**Table 3.** Electrochemical data for complexes **1**–**8** in CH_3_CN.

compound	*E*_pc_^red^ (V)	*E*_pa_ (V)	*E*_cat_ (V)	*i*_cat_ / *i*_p_	*k*_obs_ (s^−1^)	OP (V)	CE
**1**-Mn	−0.64, −2.05	0.83	−2.16^a^, −1.93^b^	15^a^, 29^b^	43^a^, 118^b^	0.7,^a^ 1.04^b^	0.97–0.50,^a^ 0.88–0.59^b^
**2**-Co	−1.55, −1.74	0.79, 1.17	−1.87	43	608	0.41	0.98–0.52
**3-**Ni	−2.13	0.62	−2.15	41	53	0.69	0.87–0.77
**4-**Cu	−1.69	0.54, 0.76	−1.85, −2.12	51	496	0.39	0.98
**5-**Mn	−0.71, −2.16	0.63	−2.39,^a^ −2.07^b^	21^a^, 31^b^	49^a^, 192^b^	0.93,^a^ 1.18^b^	0.74–0.20,^a^ 0.95–0.68^b^
**6-**Co	−1.64, −1.87	0.80, 1.27	−2.23	49	674	0.77	0.97–0.88
**7-**Ni	−2.23	0.55	−1.99, −2.27	20	80	0.53	0.90–0.26
**8-**Cu	−1.84	0.47, 0.70	−1.92, −2.14	59	686	0.46	0.67
H_2_**L^1^**	−2.31	0.99	—	—	—	—	—
H_2_**L^2^**	−2.35	0.96	—	—	—	—	—

^a^Acetic acid. ^b^Trifluoroacetic acid.

Though the complexes discussed in this work show *TOF*s in the range of 400–700 s^−1^, which is lower in comparison to few of the similar reported Schiff base complexes (see figure 1), the overpotential (OP) values are, however, on the lower side (0.39–0.8 V) for the Co, Ni and Cu complexes and higher (0.7–1.0 V) for the Mn complexes. For the reported known complexes (see figure 1), the OP values are in the range of 0.22–0.81 V and most of the measurements are in DMF or in aqueous conditions, which does not allow for a direct comparison with the catalysts discussed in this work. Complexes **1**–**8**, however, show lower OP and slightly better *TOF*s, efficiency and stability in comparison to the thiolate (di-/mono-) ligand-based complexes reported as mimics of the hydrogenase enzymes [53].

In order to get a detailed insight into the stability of the complexes and the mechanism of proton reduction, UV–visible (supplementary material, figures S14 and S16) and SEM/FESEM (supplementary material, figures S17 and S18) experiments were carried out in the absence and presence of acids. The titrations with acid were performed for selected complexes. With acetic acid, a more resolved set of UV–visible spectra with clear isosbestic points was obtained only for **2**–**4**, **6**–**8** (supplementary material, figure S14) [68,69]. For titrations with TFA as a stronger acid, small but gradual changes were seen in the UV–visible absorption spectra upon the addition of TFA to the complexes **1**–**8** (figures 8 and 9; supplementary material, figures S15 and S16). The UV absorption peaks shifted, and new peaks appeared during titration with acid, with isosbestic points at 258 (**1**); 253 (**2**); 261 (**3**); 295, 254 (**4**); 267 (**5**); 256 (**6**); 254 (**7**) and 292, 261 nm (**8**). This indicated the formation of a single product complex at all acid concentrations (figures 8 and 9; supplementary material, figures S15 and S16), as the same UV–visible spectroscopic features were observed for the complexes. Clear isosbestic points, which can be attributed to the formation of a new species, were observed for all complexes.

**Figure 8.**
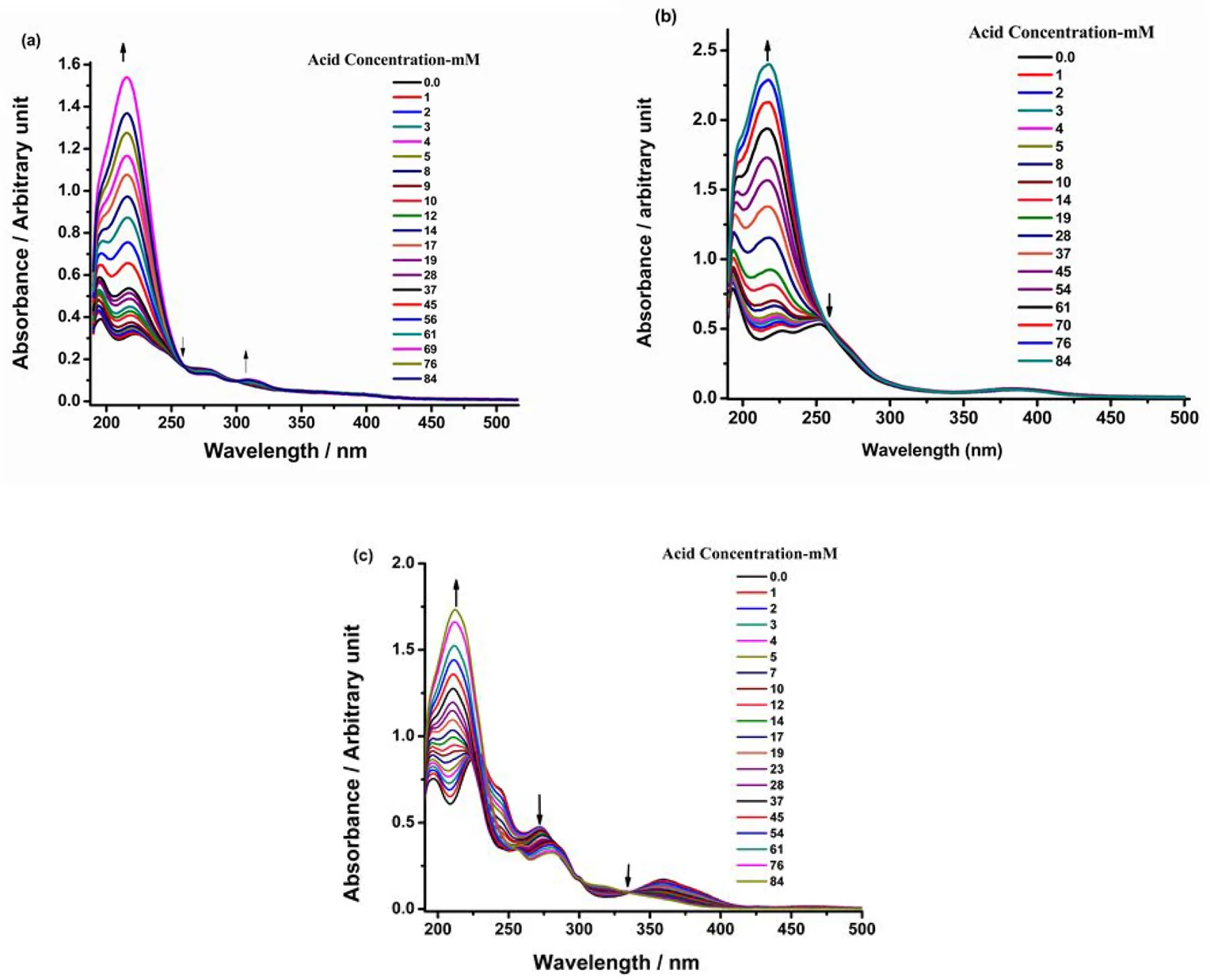
UV–visible spectra in CH_3_CN for complexes (0.01 mM) (a) [Mn^III^(salen)Cl] **1**, (b) [Co^II^(salen)] **2** and (c) [Cu^II^(salen)] **4** under different concentrations of TFA (0–84 mM). The spectral changes upon the addition of increasing amounts of acid are indicated by arrows.

**Figure 9.**
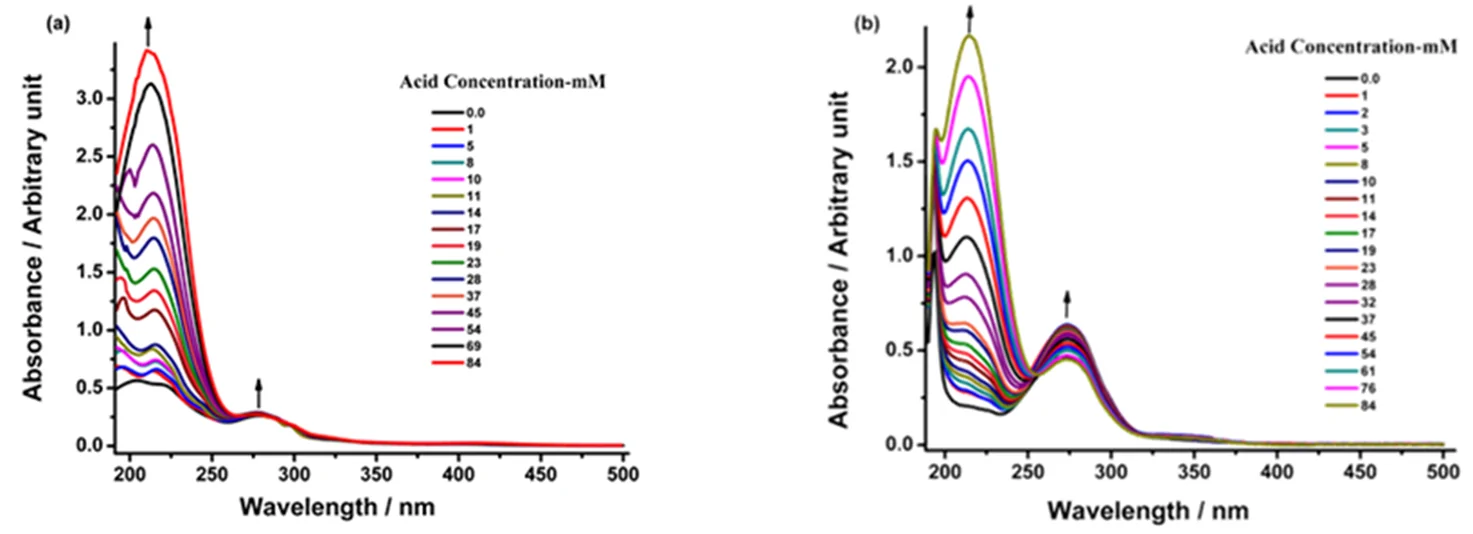
UV–visible spectra in CH_3_CN for complexes (0.02 mM) (a) [Mn^III^(CH_3_salen)Cl] **5**, and (b) [Ni^II^(CH_3_salen)] **7** under different concentrations of TFA (0–84 mM). The spectral changes upon the addition of increasing amounts of acid are indicated by arrows.

Rinse test was performed for the complexes after measuring the CVs with acid. After the CVs with acid, the glassy carbon electrode was removed from the solution, rinsed with CH_3_CN, and dipped into acetonitrile with the same amount of acid but without the catalyst. In the control experiment, no significant peaks were detected, which suggests that catalysis is homogeneous and there is no electrode deposition or complex decomposition. UV–visible spectra (for complexes **2**, Co and **8**, Cu) after bulk electrolysis also suggest that the catalysts were intact under the operating conditions and the catalytic performance was from homogeneous electrocatalysis (supplementary material, figure S19). SEM images of the catalyst measured after bulk electrolysis in the presence of acid also demonstrated the stability of the catalysts (supplementary material, figures S17 and S18).

## 4. Mechanism of the hydrogen evolution reaction

DFT calculations on solvent-phase optimized geometries at PBE0/def2-TZVP(CPCM) level of theory using ORCA 5.0.3 program were carried out to understand the HER mechanisms of all complexes. The reduction of protons (H^+^) to hydrogen (H_2_) proceeds via two tentative mechanisms: (1) two successive electrochemical processes followed by two successive chemical (protonation) processes (EECC) and (2) two alternating electrochemical and chemical processes (ECEC). We have evaluated these two mechanisms for complexes **1**–**8** (see figure 10). First, we discuss the energetics of each of the HER mechanisms and then the structural changes in each step.

***Step 1* (*One-electron Reduction*).** In both EECC and ECEC mechanisms, the first step is the reduction of LM^II^ to LM^I^ (here, L = Schiff base ligand, M = Mn, Co, Ni, Cu). The changes in Gibbs free energies are as follows: −209.8 kJ mol^−1^ (**1**), −237.6 kJ mol^−1^ (**2**), −260.5 kJ mol^−1^ (**3**), −257.9 (**4**), −216.2 (**5**), −223.6 kJ mol^−1^ (**6**), −248.3 kJ mol^−1^ (**7**) and −243.0 kJ mol^−1^ (**8**). In all complexes, this first one-electron reduction is purely metal-centred, and there is close to no spin-delocalization onto the ligand nitrogen and oxygen atoms. The calculated reduction potential values with respect to Fc/Fc^+^ agree well with experiment, indicating that this reduction process occurs in all eight cases (**1**–**8**); see above. Although in the case of complexes **1** and **5**, the first reduction is from Mn^III^ to Mn^II^, here Mn^II^ is also considered in order to allow a comparison with other species.

***Step 2a* (*Second Reduction Step, EECC*)**. In an EECC mechanism, the second reduction process is less thermodynamically favourable if metal-centred, as discussed previously. Here, the changes in Gibbs free energies are −189.4 kJ mol^−1^ (**1**), −190.0 kJ mol^−1^ (**2**), −162.4 (**3**), −169.3 (**4**), −153.4 kJ mol^−1^ (**5**), −183.4 kJ mol^−1^ (**6**), −150.7 kJ mol^−1^ (**7**) and −158.4 kJ mol^−1^ (**8**) (see scheme 2a).

**Scheme 2.**
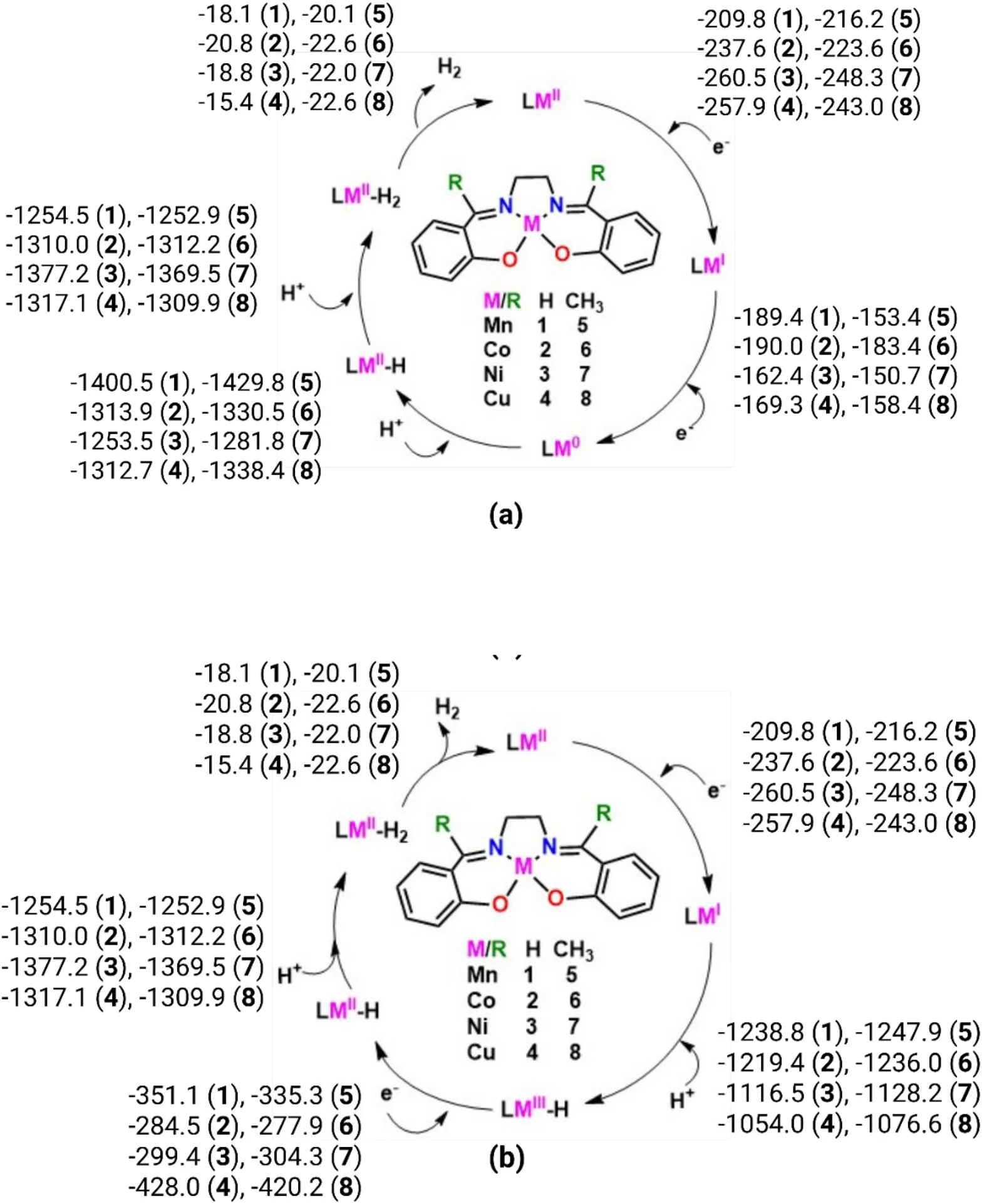
Comparison of reaction mechanisms of the hydrogen evolution reaction following an (a) EECC and (b) ECEC mechanism of complexes **1**–**8**. Gibbs free energies are given in kJ mol^−1^ with acetonitrile as the solvent.

***Step 3a* (*First Protonation, EECC*).** In an EECC mechanism, the following first protonation is fairly exothermic by −1400.5 kJ mol^−1^ (**1**), −1313.9 kJ mol^−1^ (**2**), −1253.5 kJ mol^−1^ (**3**), −1312.7 kJ mol^−1^ (**4**), −1429.8 kJ mol^−1^ (**5**), −1330.5 kJ mol^−1^ (**6**), −1281.8 kJ mol^−1^ (**7**), and −1338.4 kJ mol^−1^ (**8**) (see scheme 2a).

***Step 2b* (*First Protonation Step ECEC*).** In ECEC mechanisms protonation of the mono-reduced metals species is nearly as exothermic as protonation in EECC mechanism above; −1238.8 kJ mol^−1^ (**1**), −1219.4 kJ mol^−1^ (**2**), −1116.5 kJ mol^−1^ (**3**), −1054.0 kJ mol^−1^ (**4**), −1247.9 kJ mol^−1^ (**5**), −1236.0 kJ mol^−1^ (**6**), −1128.2 kJ mol^−1^ (**7**), and −1076.6 kJ mol^−1^ (**8**) (see scheme 2b). In either process, a metal-hydride species is formed. These numbers are in agreement with those reported for FeFe complexes, mononuclear Mn, Ru, and Fe complexes [11,14,15,40,70–72].

***Step 3b* (*Second One-electron Reduction Step, ECEC*)**. The energy released during the reduction of M^III^-H to M^II^-H in the ECEC mechanism is as follows: −351.1 kJ mol^−1^ (**1**), −284.5 kJ mol^−1^ (**2**), 299.4 kJ mol^−1^ (**3**), −428.0 kJ mol^−1^ (**4**), −335.3 kJ mol^−1^ (**5**), −227.9 kJ mol^−1^ (**6**), −304.3 kJ mol^−1^ (**7**), and −420.2 kJ mol^−1^ (**8**). The larger amount of energy released for Cu compared to Mn, Co and Ni indicates a facile reduction of Cu in this step (see scheme 2b). But this trend is reversed in the case of protonation of M^I^ to M^III^-H and is found to be −1065.1 (−1098.8) kJ mol^−1^ for Cu **3** (**6**), which is the least compared to other Co and Ni species (see scheme 2b).

***Step 4* (*Second Protonation*)**. Protonation of the axial metal-coordinated hydride (M^II^-H) of Co, Ni and Cu leads to the weakly interacting hydrogen (H_2_) intermediate that is common in either HER. Here, a new strong covalent H-H bond is formed. The protonation energies for hydride complexes **1**–**8** are −1254.5 kJ mol^−1^ (**1**), −1310.0 kJ mol^−1^ (**2**), −1377.2 kJ mol^−1^ (**3**), −1317.1 kJ mol^−1^ (**4**), −1252.9 kJ mol^−1^ (**5**), −1312.2 kJ mol^−1^ (**6**), −1369.5 kJ mol^−1^ (**7**), and −1309.9 kJ mol^−1^ (**8**).

Binding of H_2_ is weak and release of molecular hydrogen is thermodynamically possible by −18.1 kJ mol^−1^ (**1**), −20.8 kJ mol^−1^ (**2**), −18.8 (**3**), −15.4 (**4**), −20.1 kJ mol^−1^ (**5**), −22.6 kJ mol^−1^ (**6**), −22.0 kJ mol^−1^ (**7**) and −22.6 kJ mol^−1^ (**8**) to complete the HER cycle. The overall thermodynamics for all the complexes are thus negative, which suggests the feasibility of the hydrogen evolution reaction in these complexes.

### 4.1. Structural changes in EECC and ECEC hydrogen evolution reaction mechanisms

Here, we explain how the structural parameters change during the EECC and ECEC mechanisms. As the first reduction is metal-centred, the bond lengths of M–O increase by 0.02/0.01 Å (**1**, **5**), 0.05 Å (**2**, **6**), 0.10 Å (**3**, **7**) and 0.10 Å (**4**, **8**), while M–N bond lengths decrease slightly for **1**, **5** (Mn, −0.03 Å) and **2**, **6** (Co, −0.04/-0.03 Å) and increase for **3**, **7** (Ni, 0.04/0.05 Å) and **4**, **8** (Cu, 0.04 Å). After the second reduction step in an EECC mechanism, the M–O bond distances further increase by 0.02 Å (**1**, **5**), 0.00/−0.06 Å (**2**, **6**), −0.01/−0.02 Å (**3**, **7**) and 0.03/0.00 Å (**4**, **8**), while M–N distances only change marginally by −0.01/0.00 Å (**1**, **5**), 0.01/0.07 Å (**2**, **6**), −0.02 Å (**3**, **7**) and 0.01/0.00 Å (**4**, **8**). This change indicates that the second reduction affects the metal–ligand coordination more than the first reduction step discussed above. If the reduced metal is protonated to give the corresponding metal hydride the M–O bond lengths decrease by −0.03 Å (**1**, **5**), −0.02 Å (**2**, **6**), −0.05 Å (**3**, **7**) and −0.13/−0.11 Å (**4**, **8**), while M–N bond lengths increase as 0.03 Å (**1**,**5**), 0.05 Å (**2**,**6**), 0.02/0.01 Å (**3**,**7**) and −0.07/0.02 Å (**4**,**8**). These large variations in the structural parameters in the M–O bond lengths of complexes **4** and **8** indicate that the ECEC path is hardly possible, and the reactions proceed rather via an EECC instead of an ECEC mechanism. Similarly, if a proton is added after the second reduction step in an EECC pathway the M–O bond lengths change by 0.00 Å (**1**, **5**), 0.03/0.08 Å (**2**, **6**), −0.03/−0.01 Å (**3**, **7**) and −0.10/0.12 Å (**4**, **8**), while M–N bond lengths vary by 0.06/0.05 Å (**1**, **5**), 0.03/−0.02 Å (**2**, **6**), 0.21/0.22 Å (**3**, **7**) and 0.02/0.04 Å (**4**, **8**). These large variations in the structural parameters of M–N bond lengths of complexes **1**, **3**, **5** and **7** suggest the non-feasibility of the formation of a second reduced species, and the mechanism proceeds via ECEC in these cases. Similarly, the metal-hydride bonds (M–H) after first protonation in the ECEC mechanism are found to be 1.52 Å (**1**, **5**), 1.43 Å (**2**, **6**), 1.51 Å (**3**, **7**) and 1.45/1.47 Å (**4**, **8**). The differences in the M–H bonds after first protonation in EECC (M^II^-H) and ECEC (M^III^-H) mechanisms are as follows: 0.29/0.30 Å (**1**, **5**), 0.22/0.24 Å (**2**, **6**), −0.03 Å (**3**, **7**) and 0.13/0.12 Å (**4**, **8**). These differences in M–H bond lengths are due to the addition of an extra electron to M^III^-H to form M^II^-H in the ECEC mechanism. The minute variation in Ni–H and large variation in Ni–N distances in complexes **3** and **8** suggest an electron delocalization onto the salen ligand. As discussed above, protonation and H_2_ release following the second protonation step are thermodynamically very favourable (see scheme 2). The second protonation (M…H_2_) bond length ranges from 5.0 to 13.0 Å, which suggests a facile and quick release of hydrogen in all the cases.

### 4.2. Comparative analysis of ECEC and EECC mechanisms

In an ECEC mechanism, the Gibbs free energy change upon reduction of M^III^-H to M^II^-H is larger for Cu compared to Mn, Ni and Cu, which indicates that the reduction of Cu will be more feasible (see figure 10a,b). But this trend is reversed in the case of protonation events M^I^ to M^III^-H and is found to be −1054.0 (−1076.6) kJ mol^−1^ for Cu **4** (**8**), which is the lowest compared to other Mn, Co and Ni species (see scheme 2b). From this, it may be possible that the Cu complexes (**4**, **8**) may follow the EECC mechanism. In general, the two consecutive electron transfers lead to the high-energy two-electron reduced intermediate, but as seen in figure 10, the partial delocalization of electron density over the ligand favours the EECC mechanism for Cu complexes over the other complexes (Mn/Co/Ni).

**Figure 10.**
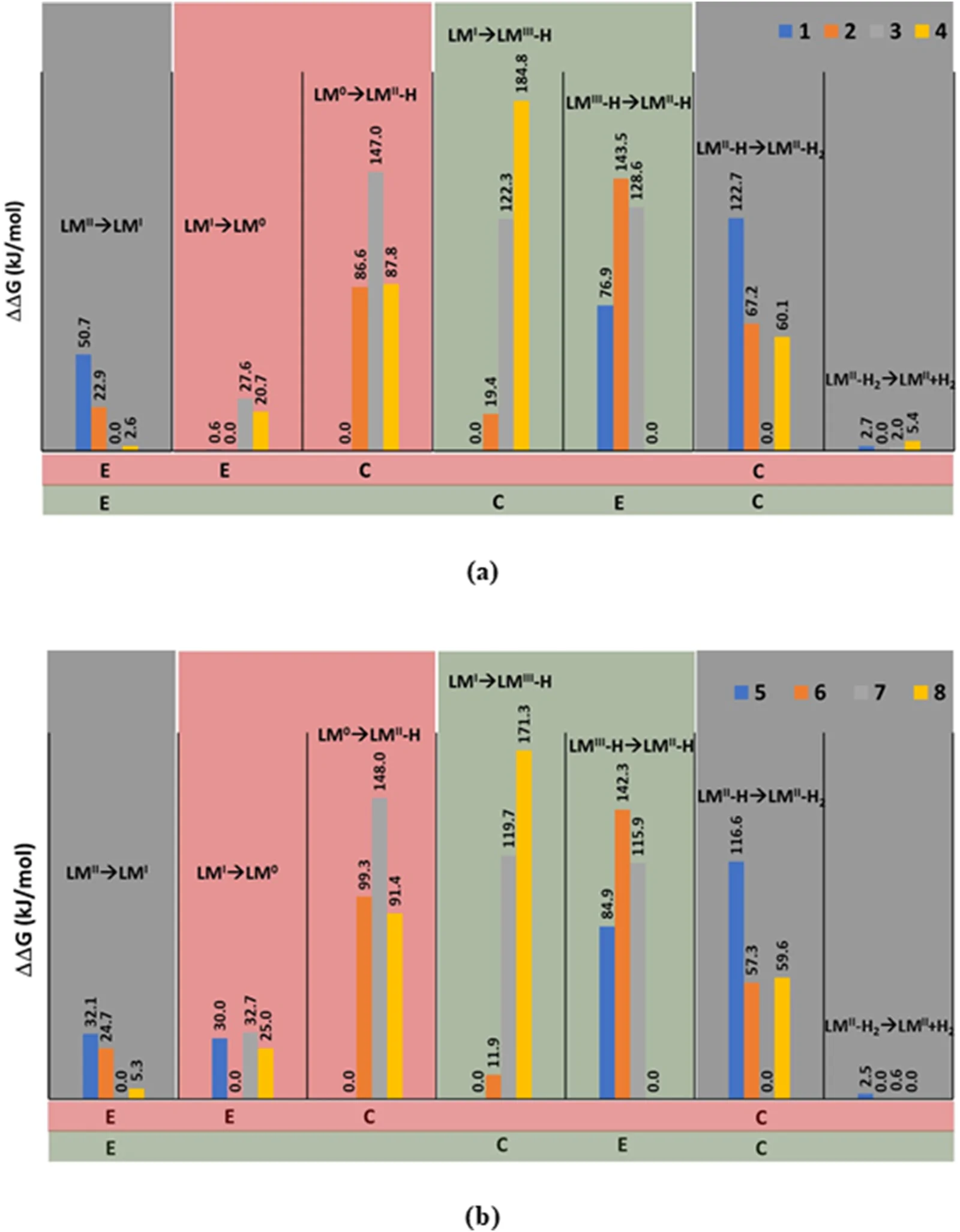
Relative Gibbs free energy changes (Δ*G*, kJ mol^−1^) for the electrochemical (E) and chemical (C) processes of (a) complexes Mn (**1**), Co (**2**), Ni (**3**), Cu (**4**) (L = **L^1^**) and (b) complexes Mn (**5**), Co (**6**), Ni (**7**), Cu (**8**) (L = **L^2^**). The possible EECC and ECEC mechanisms are given in red and green fields, respectively.

The first reduction step is energetically less prominent for complexes **1** and **5** compared to the other metal complexes (see figure 10), which is in agreement with the experimental observation of a slow reactivity. However, the large proton affinity of the Mn^I^ species promotes the ECEC mechanism.

The Co complexes (**2**, **6**) follow an EECC mechanism, which is different from the literature-reported ECEC mechanism [73,74]. The EECC mechanism is evident from the low second one-electron reduction potentials compared to the other complexes; these low values are attributed to the partial reduction of the ligand instead of being metal-centred (see supplementary material, figure S21). Also, the proton affinity of Co complexes is similar to that of the Cu complexes for the subsequent protonation steps (see figure 10).

The Ni complexes (**3**, **7**) can only undergo an ECEC mechanism, since the second one-electron reduction is the least favoured and also the subsequent protonation energies among all the complexes. For Ni complexes, a high overpotential is required to switch these mechanisms, which is clearly visible in the experimental observations. The complex **3** crosses the barrier at 0.67 V overpotential to proceed with the reaction via the EECC mechanism, while the complex **7** proceeds with the reaction via the ECEC mechanism at 0.53 V overpotential and shows slow reactivity.

## 5. Conclusion

In conclusion, we studied electrochemical hydrogen evolution employing cheap and earth-abundant Schiff base compounds as catalysts under a homogeneous system in organic solvents in the presence of a proton source. The observation of high current enhancement and lowering of the overpotential of proton reduction, which is indicative of quantitative hydrogen production, suggests the potential role of the compound as a promising HER electrocatalyst. Ongoing efforts are focused on modifying Schiff base ligands and related complexes to further facilitate new functional studies, with emphasis on chemistry relevant to sustainable energy cycles. From the DFT studies, we observe different reaction mechanisms for Mn, Co, Ni, Cu Schiff base complexes. An ECEC mechanism appears plausible for **1**, **5** (Mn) and **7** (Ni), while an EECC mechanism is seen for the **2**, **6** (Co), **3** (Ni) and **4**, **8** (Cu) complexes. The EECC mechanism is related to the high reactivity of the doubly reduced species from complexes **2**, **6** (Co), **3** (Ni) and **4**, **8** (Cu), while a slower reactivity can be related to the ECEC mechanism. The overall thermodynamics of the hydrogen generation indicate the feasibility of the reaction. The design of efficient HER evolution catalysts requires a deep understanding of the electronic structure of transition metal–ligand interactions, the reducibility of the complexes and the p*K*_a_ of the metal-hydride species. It is the balance of all these properties that determines the reactivity and mechanism of electrochemical proton reduction.

## 6. Methods

### 6.1. Materials

All chemicals—salicylaldehyde (99.5%), o-hydroxy acetophenone (95%), ethylenediamine (99.5%), NiCl_2_.6H_2_O (99%), CoCl_2_.6H_2_O (99%), CuCl_2_.2H_2_O (99%), MnCl_2_.4H_2_O, Mn(OAc)_2_.4H_2_O (99%) and solvents—were purchased from Sigma-Aldrich and were used without further purification.

### 6.2. Synthesis of the Schiff base ligands and complexes 1–8

The symmetric Schiff base ligands *N*,*N*′-bis(salicylidene)ethylenediamine (H_2_**L^1^**) and *N*,*N*-bis(*o*-hydroxyacetophenone)ethylenediamine) (H_2_**L^2^**) and the metal complexes **1**–**8** were synthesized as reported in the literature (see supplementary material) [37,38].

### 6.3. Physical measurements

The NMR spectra of the ligands and the complexes were recorded at 25°C in DMSO-*d*_6_ using a Bruker 400 MHz instrument. The infrared spectra were collected using a PerkinElmer FTIR spectrometer (4000–400 cm^−1^) fitted with a Pike ATR. The UV–visible spectra for complexes were recorded in CH_3_CN on a PerkinElmer Lambda spectrophotometer. Mass spectra were recorded with a quadrupole time-of-flight mass spectrometer with ESI and APCI sources (Agilent G6530AA [LC-HRMS-Q-TOF]). Elemental (C, H, and N) analyses were performed on a Leco TruSpec Micro CHNS analyser. Field emission scanning electron microscopy recorded with a JEOL (JSM6610LV) with energy dispersive spectrometry (FESEM/EDS) was used for morphological evaluation. The complexes **1**–**8** were recrystallized from ethanol to get X-ray diffraction-quality crystals of the complexes. X-ray diffraction-quality crystals were obtained for complexes **1**, **3**, **4**, **8** from ethanol solvent, the data for which matched with those reported in the literature [46–50].

### 6.4. Electrochemistry

Cyclic voltammetry measurements were conducted in CH_3_CN with 0.1 M [N(*n*-Bu_4_)][PF_6_] (tetrabutylammonium hexafluorophosphate) as the supporting electrolyte that was dried in vacuum at 383 K. An Autolab potentiostat with a Nova 1.1 electrochemical interface was used to carry out the cyclic voltammetry measurements. A glassy carbon disc (diameter 3 mm, freshly polished) was chosen as a working electrode for cyclic voltammetry. Platinum wire was used as the counter electrode. The reference electrode was a non-aqueous Ag/Ag^+^ electrode (CH Instruments, 0.01 M AgNO_3_ in CH_3_CN). All the potentials are quoted against the ferrocene/ferrocenium couple (Fc/Fc^+^); ferrocene was added as an internal standard at the end of the experiments. For electrochemical measurements, all solutions were prepared from dry CH_3_CN (Sigma-Aldrich, spectroscopic grade, dried with molecular sieves (MS, 3 Å)). Bulk electrolysis experiments were performed in acetonitrile under constant argon purging and continuous stirring at a particular potential.

### 6.5. Computational details

All the geometry optimization was performed using the ORCA 5.0.3 program [71,72,75,76]. The GGA BP86 functional is used for the calculation of electrochemical potential values of all the complexes with the def2-TZVP basis set for all the atoms and split-RI-J approximation [73,74,77–79]. Scalar relativistic effects were taken into account with the zero-order regular approximation (ZORA) [80]. All optimizations are solvent-phase optimizations using the Conductor-like Polarizable Continuum Model (CPCM) with acetonitrile solvent [81,82]. The redox potential of all the complexes has been calculated relative to the ferrocene/ferrocenium (Fc/Fc^+^) reference electrode in acetonitrile solvent [83]. The HER mechanism is computed at PBE0/def2-TZVP level of theory with similar settings to those discussed above [84].

## Supplementary Material



## Data Availability

Supplementary material is available online [85].
