# Supplementary material for: New use of mononuclear Mn/Co/Ni/Cu complexes of symmetric N2O2-type Schiff base as hydrogen-evolving catalysts
Source: R Soc Open Sci. 2026 Oct 14;13(10):260074. doi: 10.1098/rsos.260074 (PMC13682433; doi:10.1098/rsos.260074)
Supplement: Supplementary file 1 [file rsos.260074.zip › rsos.260074/rsos260074_si_001.docx]

**New Use of Mononuclear Mn/Co/Ni/Cu-Complexes of Symmetric N_2_O_2_-type Schiff-Base Hydrogen Evolving Catalysts**

Fatimah Ali Hussein^1^, Ravi Kumar^2,3^, Matthias Stein^2*^ and Sandeep Kaur-Ghumaan^1^*

^1^Inorganic & Bio-Inorganic Research Laboratory

Department of Chemistry, University of Delhi (North Campus), Delhi 110007, India

^2^ Molecular Simulations and Design Group, Max Planck Institute for Dynamics of Complex Technical Systems, Magdeburg, Germany

^3^Department of Chemistry, University of Delhi (North Campus), Delhi 110007, India

*Email: [skaur@chemistry.du.ac.in](mailto:skaur@chemistry.du.ac.in%20); [matthias.stein@mpi-magdeburg.mpg.de](mailto:matthias.stein@mpi-magdeburg.mpg.de)

**S****upporting Information**

**Synthesis of Schiff base complexes 1-8**

The Salen and CH_3_Salen ligands were synthesized following the established protocols using ethylenediamine and the corresponding salicylaldehyde, and o-hydroxy acetophenone precursors (1:2 ratio) in absolute ethanol (refluxed for 1-3h).^S1-S2^ The complexes were synthesized by general methods. The prepared Schiff base ligands were subsequently refluxed with stoichiometric amounts of M^II^-salts (chloride or acetate salts) in ethanol (EtOH) (methanol (MeOH) can also be used) to yield the desired complexes as reported in literature - [Mn^III^(Salen)Cl] **1**,^S3-S5^ [Co^II^(Salen)] **2**,^S6^ [Ni^II^(Salen)] **3**,^S7-S9^ [Cu^II^(Salen)] **4**,^S7-S9^ [Mn^III^(CH_3_Salen)Cl] **5**,^S3-S5^ [Co^II^(CH_3_Salen)] **6**,^S10-S11^ [Ni^II^(CH_3_Salen)] **7**^S11^ and Cu^II^(CH_3_Salen)] **8**.^S12^

**Figure S1.**	UV-Vis spectra for complexes (a) **1-4** (0.01 mM), (b) **5-8** (0.02 mM) and (c) ligands (H_2_**L^1^** and H_2_**L^2^**) (0.05 mM) in CH_3_CN.

**Figures S2.** FESEM images for complexes **1-4** before catalysis.

**Figures S3.** FESEM images for complexes **5-8** before catalysis.

**Figure S4.**	CVs (reduction) for complexes **1-8** in the absence of acidic media in CH_3_CN at a scan rate of 0.1 Vs^−1^ in CH_3_CN.

**Figure S5.**	CVs (oxidation) for complexes **1-8** in the absence of acidic media in CH_3_CN at a scan rate of 0.1 Vs^-1^.

**Figure S6.** Cyclic voltammograms (reduction, top and oxidation, bottom) for ligands H_2_**L^1^** (▬) and H_2_**L^2^** (▬) (1 mM) in CH_3_CN at a scan rate 0.1 Vs^‒1^.

**Figure S7.**	Randles–Sevcik plots (reduction) for complexes (1 mM) (a) **1** (■), **5** (●), (b) **2** (■), **6** (●), (c) **3** (■), **7** (●) and (d) **4** (■), **8** (●) in CH_3_CN.

**Figure S8.**	CVs for complexes (1 mM) (a) **1** and (b) **5** in the absence (**- - -**) and with increasing (⎯) concentrations of AcOH (1-10 mM, **1**; 1-50 mM, **5**) in CH_3_CN at a scan rate of 0.1 Vs^‒1^.

**Figure S9.**	CVs for CH_3_CN (**- - -**), CH_3_CN/10 mM AcOH (**▬**) and CH_3_CN/10 mM AcOH/1 mM complex **1** (**▬**) / **5** (**▬**) at a scan rate of 0.1 Vs^‒1^.

**Figure S10.**	CVs for (a) CH_3_CN (- - -), CH_3_CN / 50 mM TFA (**▬**), CH_3_CN / 50 mM TFA / 1 mM complex **1** (**▬**) and (b) CH_3_CN (- - -), CH_3_CN/20 mM TFA (**▬**) and CH_3_CN/20 mM TFA/1 mM complex **5** (**▬**) at a scan rate of 0.1 Vs^−1^.

**Figure S11.** CVs for CH_3_CN (- - -), CH_3_CN / 50 mM AcOH (**▬**) and 1 mM complexes (a) **2** (**▬**) and **6** (**▬**), (b) **3** (**▬**) and **7** (**▬**) and (c) **4** (**▬**) and **8** (**▬**) in CH_3_CN in the presence of 50 mM AcOH at a scan rate of 0.1 V s^−1^.

**Figure S12.**	Plots of (a) *i*_cat_ */i*_p_ and (b) *k*_obs_/s^-1^ vs. [AcOH] for complexes (1 mM) **1** (black), **2** (red), **3** (green) and **4** (blue), (c) *i*_cat_ */i*_p_ vs. and (d) *k*_obs_/s^-1^ vs. [AcOH] for complexes (1 mM) **5** (black), **6** (red), **7** (green) and **8** (blue) in CH_3_CN at 0.1 Vs^−1^.

**Figure S13.**	Plots of (a) *i*_cat_ */i*_p_ and (b) *k*_obs_/s^-1^ vs. [TFA] for complexes (1 mM) **1** (black), **5** (red) in CH_3_CN at 0.1 Vs^-1^.

**Figure S14.**	UV-Vis spectra for complexes (0.01 mM) (a) **2** (**▬**), **3** (**▬**) and **4** (**▬**) before catalysis and **2** (- - -), **3** (- - -), **4** (- - -) in the presence of 50 mM of AcOH and (b) **6** (**▬**), **7** (**▬**) and **8** (**▬**) before catalysis and **6** (- - -), **7** (- - -), **8** (- - -) in the presence of 50 mM of AcOH in CH_3_CN.

**Figure S15.**	UV-Vis spectra in CH_3_CN for complex [Ni^II^(Salen)] **3** (0.01 mM) with different concentrations of TFA (0-84 mM). The spectral changes upon the addition of increasing amounts of acid are indicated by arrows.

**Figure S16.**	UV-vis absorption spectra in CH_3_CN for 0.02 mM complexes (a) [Co^II^(CH_3_Salen)] **6** and (b) [Cu^II^(CH_3_Salen)] **8** with different concentrations of TFA (0-84 mM). The spectral changes upon the addition of increasing amounts of acid are indicated by arrows.

**Figure S17.** FESEM images for complexes **1-4** after catalysis.

**Figure S18.** FESEM images for complexes **5-8** after catalysis.

**Figure S19.** UV–Vis spectra for 0.01 mM complexes (a) **2** (Co) and (b) **8** (Cu) before and after bulk electrolysis.

**Figure S20.**	Tafel plots of log *k_obs_* vs. overpotential (AcOH) for complexes (1 mM) (a) **1** (■), **2** (●), **3** (▲) and **4** (▼), (b) **5** (■), **6** (●), **7** (▲) and **8** (▼) in CH_3_CN at 0.1 Vs^‒1^.

**Figure S21.**	Spin-density plots of the optimized structures of doubly reduced states Mn^II^-L^●−^ (**1**, **5**) (a, b) and Co^I^-L^●−^ (**2**, **6**) (c, d) Schiff base complexes in the broken symmetry state at contour values of 0.01 a.u.

**References**

S1.	Abdalla EM, Abdel Rahman LH, Abdelhamid AA, Shehata MR, Alothman AA, Nafady A. Synthesis, characterization, theoretical studies, and antimicrobial/antitumor potencies of salen and salen/imidazole complexes of Co(II), Ni(II), Cu(II), Cd(II), Al(III) and La(III). Applied Organometallic Chemistry. 2020 Nov; 34(11):e5912. (<https://doi.org/10.1002/aoc.5912>)

S2.	Hamil AM, Abdelkarem M, Hemmet M, El-ajaily MM. Synthesis of a new schiff base: 2-[2-(e)-(2-hydroxyphenyl)-ethylidene]aminoethyl)ethanimidoyl]phen. International Journal of ChemTech Research. 2012 Apr;4(2):682-5. ([https://sphinxsai.com/2012/chemAJ/CHEM/CT=31[682-685]AJ12.pdf](https://sphinxsai.com/2012/chemAJ/CHEM/CT=31%5b682-685%5dAJ12.pdf))

S3.	Idage BB, Idage SB, Kasegaonkar AS, Jadhav RV. Ring opening polymerization of dilactide using salen complex as catalyst. Materials Science and Engineering: B. 2010 Apr 15;168(1-3):193-8. (<https://doi.org/10.1016/j.mseb.2009.10.037>)

S4.	Meftah S, Yazdanparast R. Synthesis and antioxidant activities of [5-Fluoro N, N’-Bis(salicylidene)ethylenediamine] and [3, 5-Fluoro N, N’-Bis(salicylidene) ethylenediamine] manganese(III) complexes. (<https://www.sid.ir/paper/593964/en>)

S5.	Neves ES, Fernandes C, Horn Jr A. Study of cyclohexane and methylcyclohexane functionalization promoted by manganese (III) compounds. Inorganics. 2023 Mar 3;11(3):105. (<https://doi.org/10.3390/inorganics11030105>)

S6.	Kumar DN, Garg BS. Some new cobalt(II) complexes: synthesis, characterization and thermal studies. Journal of thermal analysis and calorimetry. 2002 Aug;69(2):607-16. (<https://doi.org/10.1023/A:1019976226610>)

S7.	da Silva MD, Gonçalves JM, Silva AL, Oliveira PC, Schröder B, da Silva MA. Molecular thermochemical study of Ni(II), Cu(II) and Zn(II) complexes with N, N′-bis (salicylaldehydo) ethylenediamine. Journal of Molecular Catalysis A: Chemical. 2004 Dec 15;224(1-2):207-12. (<https://doi.org/10.1016/j.molcata.2004.09.008>)

S8.	Salma U, Rana MS, Ferdous J, Hossen MF, Zahan MK, Zamir R, Asraf MA. Metal-salen complexes: structural characterization and antibacterial activity. Asian Journal of Chemical Sciences. 2022 Dec 31;12(4):45-56. (<https://hal.science/hal-05210502/>)

S9.	Nworie FS. Bis(salicylidene)ethylenediamine (salen) and bis(salicylidene) ethylenediamine-metal complexes: From structure to biological activity. J. Anal. Pharm. Res. 2016 Dec 15;3(6):1-0. (<https://doi.org/10.15406/JAPLR.2016.03.00076>)

S10.	Pui A, Mahy JP. Synthesis, characterization and catalytic activity of halo-methyl-bis (salicylaldehyde) ethylenediamine cobalt (II) complexes. Polyhedron. 2007 Aug 6;26(13):3143-52. (<https://doi.org/10.1016/j.poly.2007.02.030>)

S11.	Gupta KC, Sutar AK. Polymer anchored Schiff base complexes of transition metal ions and their catalytic activities in oxidation of phenol. Journal of Molecular Catalysis A: Chemical. 2007 Jul 2;272(1-2):64-74. (<https://doi.org/10.1016/j.molcata.2007.03.025>)

S12.	Pui A. Mechanism of oxidation of 2, 6‐di‐tert‐butyl‐phenol with molecular oxygen, in presence of some new bis (di‐halogenoα‐methyl salen) copper(II) complexes. Synthesis and Reactivity in Inorganic, Metal-Organic and Nano-Metal Chemistry. 2006 Aug 1;36(7):523-7. (<https://doi.org/10.1080/15533170600862259>)
